# Bacterial Diversity in Leaf-Cutter Ant Species: Host-Microbe Interactions and Environmental Effects

**DOI:** 10.1007/s13744-026-01386-7

**Published:** 2026-06-11

**Authors:** Alexandra Gianaris, Manuela O. Ramalho, Amanda A. de Oliveira, Maria Santina de Castro Morini, Cintia Martins, Odair C. Bueno

**Affiliations:** 1 Dept of Biology, West Chester Univ, West Chester, PA USA; 2 Dept of General and Applied Biology, São Paulo State Univ, Rio Claro, SP Brazil; 3 Lab de Mirmecologia Do Alto Tietê (LAMAT), Univ de Mogi das Cruzes, Mogi das Cruzes, São Paulo Brazil; 4 Dept of Biology, Univ Federal Do Delta Do Parnaíba, Parnaíba, Piauí Brazil

**Keywords:** Bacterial diversity, Bacterial communities, Microbial ecology, Microbiology, Mutualist, Symbiont

## Abstract

Historically, studies have sought to identify host-specific factors in host-microbe interactions as a means of understanding evolutionary success. The genus *Atta*, comprising leaf-cutter ants, is native to the southern Neotropics and obligately mutualistic with cultivated fungi. It hosts a remarkably diverse range of bacterial communities, yet this variability remains poorly understood. Using high-throughput amplicon sequencing of the 16S rRNA genes of the whole worker, we showed significant difference between the bacterial communities among 4 dominant *Atta* species: *Atta sexdens* (Linnaeus), *Atta laevigata* (Smith), *Atta capiguara* (Gonçalves), and *Atta bisphaerica* (Forel). We also discovered significant differences in bacterial communities from laboratory conditions, pesticide treatment, and the fungal garden symbiont of *Atta sexdens*. Surprisingly, bacterial communities of *Atta sexdens* kept in the laboratory were not significantly different from pesticide-treated *Atta sexdens*, laying the groundwork for potential refinement of standard research methods.

**Supplementary Information:**

The online version contains supplementary material available at 10.1007/s13744-026-01386-7.

## Introduction

Microorganisms influence all living organisms, playing a pivotal role in global ecosystem function (Wagg et al. 2019). Microbial communities can be exceptionally complex, exhibiting a vast range of functions from nutrient and carbon cycling, to understanding host health and phenotypes (Wagg et al. 2019; Chevrette et al. 2022). To understand host-microbe interactions and microbe function, the composition of bacterial communities must be explored. Bacterial communities influence organism fitness (Vandenkoornhuyse et al. 2015), pathogen protection (Bulgarelli et al. 2012), nutrient intake (Bulgarelli et al. 2012), environmental distress protection (Walsh et al. 2021; Loftus et al. 2021), and global adaptability (Vandenkoornhuyse et al. 2015) in various biogeochemical cycles. Host factors such as species, rearing method, and pesticide treatment are variable. Exploring the composition of bacterial communities is crucial to allow for future investigation into the impact of these host-microbe interactions. This paves the way towards understanding coevolution of microorganisms comprising bacterial communities (Ogunrinola et al. 2020).

Ants hold a dominant ecological role in nearly all global terrestrial environments (Garrett et al. 2016; Wheeler 1910; Hölldobler and Wilson 1990). The Formicidae family of insects is incredibly vast and diverse, housing all ants (Bolton 2026). Associations between ants and microorganisms are ancient (Vieira et al. 2017). This has led to the extensive study of ant bacterial communities. Functions of some bacteria have even been established as vital to survival, such as necessary metabolization of lipids, sulfur, and nitrogen in the *Camponotus* and *Cephalotes* genera (Degnan et al. 2005; Wernegreen et al. 2009; Ramalho et al. 2017, 2018; Hu et al. 2018; Ramalho et al. 2020a, b; Ramalho and Moreau 2023). Further research is needed to unravel the immense diversity of the bacterial interactions within the 350 valid genera of the Formicidae family (Bolton 2026).

Commonly known as leaf-cutter ants, *Atta* spp. are fungivores, and are characterized by their fungus-growing worker behavior. *Atta* workers cut disc-like fragments of leaves and other foliage in the environment for use as substrate to cultivate their mutualistic fungi, *Leucoagaricus gongylophorus*. These gardens produce specialized hyphal swellings, which serve as a source of food for their immatures (Aylward et al. 2013; Garrett et al. 2016). To construct their nests, *Atta* transport colossal amounts of soil (Forti et al. 2020), providing potential insight into the functions and origins of their bacterial communities. Few studies have explored the composition of bacterial communities associated with *Leucoagaricus gongylophorus* and *Atta* workers (Suen et al. 2010; Aylward et al. 2012, 2014; Khadempour et al. 2020). This behavior is deeply impactful to the surrounding environment; *Atta* have been classified as pests (Fowler et al. 1989; Forti et al. 2020) and have caused estimated billions of dollars of damage via attack and severe defoliation of green herbivory in their home range (Montoya-Lerma et al. 2012; Ramalho et al. 2020a, b). As a result, large quantities of pesticides, such as sulfuramid, are commonly used against leaf-cutting ants (Bejarano et al. 2019; Della Lucia & Amaral 2020).

There are 15 species of *Atta* exclusively found in the Neotropical and Nearctic regions, nine of which are present in Brazil (Forti et al. 2020; Barrera et al. 2022). The São Paulo State of Brazil hosts four native species of *Atta*: *Atta bisphaerica*, *Atta capiguara*, *Atta laevigata*, and *Atta sexdens*. These four species within the *Atta* genus are prevalent in widespread distribution across Brazil, and prove ideal to compare their host bacterial communities due to their vast geographic distribution (Forti et al. 2020).

Host bacterial communities may also be significantly impacted by the rearing environment of hosts. Lab colonies are commonly used to study ants, which may significantly affect the composition of ant bacterial communities. Surprisingly, the difference between the bacterial communities of lab colonies of *Atta* versus samples reared in different environments has not yet been explored. Therefore, it is tremendously important to explore the importance of the environments in which samples are reared. 

Although some genera of ants present strong associations to hosting a core (50%) bacterial community relatively undisturbed in hosts (Degnan et al. 2005; Wernegreen et al. 2009; Ramalho et al. 2017, 2018; Zhukova et al. 2017; Hu et al. 2018; Ramalho et al. 2020a, b; Kelleher and Ramalho 2025a, 2025b), other studies have shown the presence of bacterial communities in ants without any core taxa and higher variability (Ramalho et al. 2020a, b; Martins and Moreau 2020). The expansive diversity across the composition and abundance of the bacterial communities of *Atta* suggests that the communities within this genus may be transient, or more variable. This suggests that bacteria have a temporary association with their hosts rather than an intrinsic presence in bacterial communities (Hu et al. 2018; Moreau 2020; Duplais et al. 2021; Kelleher and Ramalho 2025a, b). This hypothesis is the main driver of this study as we seek to understand the variability within the bacterial communities of *Atta*. An enhanced understanding of these interactions would not only provide a groundbreaking distinction to *Atta* but also lay the foundation for interpreting the factors that shape and drive distinct bacterial community variability.

This study aims to further the understanding of the bacterial communities of *Atta* species, and provide a foundation for future research to be conducted investigating host-microbe interactions and the role in the evolutionary success of leaf-cutting ant species in the southern Neotropics. Specifically, we investigate the associated bacterial communities in four predominant leaf-cutting ant species: *Atta sexdens*,* Atta laevigata*,* Atta capiguara*, and* Atta bisphaerica*. Despite occupying similar ecological niches and being allopatric, these species may exhibit distinct bacterial communities. Using 16S rRNA amplicon sequencing, we seek to elucidate whether different species rely on different bacterial communities, providing insights into the evolution of these ants in their respective environments and conditions. Our study not only sheds light on the vast and variable microbial diversity within these ant species individually, but also explores potential interconnections of these bacterial communities. We address several key questions: Is there a difference among these *Atta* species in their bacterial community composition? Is there a difference between associated bacteria of *Atta sexdens* collected in their natural habitat to *Atta sexdens* reared in the lab? Is there a difference between pesticide-treated and non-pesticide-treated *Atta sexdens* reared in the lab in terms of their associated bacteria? Finally, is there a difference between the bacterial communities of the *Atta* species and their symbiotic fungi? By answering these questions, we aim to provide a comprehensive understanding of the role of bacterial communities in the evolution and ecology of these leaf-cutting ants.

## Methods

### Sample collection

All samples consisted of one whole worker ant, totaling 39 workers of *Atta* spp. collected from the state of São Paulo, Brazil, in their natural habitats. Colonies sampled were 200 m or further apart. In terms of species classification, 27 workers were identified as *Atta sexdens*, three as *Atta laevigata*, three as *Atta capiguara*, and three as *Atta bisphaerica*. Samples were collected from nine different *Atta sexdens* colonies, and one colony each of *Atta laevigata*, *Atta capiguara*, and *Atta bisphaerica*. Additionally, three *Atta sexdens* workers were kept in the laboratory, and three isolated samples of *Leucoagaricus gongylophorus* fungal garden were included (Nogueira et al. 2022).

Laboratory samples were maintained following Nogueira et al. (2022) in open nest area containers to limit colony disturbance. The *Leucoagaricus gongylophorus* samples were collected from one colony of *Atta sexdens*. A portion of approximately 20 g of the garden was removed with sterile tweezers and placed in a 100-ml polypropylene plastic pot, then placed in a freezer for a duration of 5 min. Ants were then removed, leaving the fungus garden to be separated into three aliquots of equal mass, with one aliquot comprising one sample.

Finally, three adult colonies of *Atta sexdens* maintained in the laboratory (see Nogueira et al. 2022) were provided with pelleted bait containing 2 g of 0.03% sulfuramid over a span of 3 months to evaluate the pesticide effect on their bacterial communities from long-term exposure. Workers from each colony were randomly sampled to assess impact of long-term exposure on microbiota. The taxonomy of all ant samples was identified to a species level using Mackay and Mackay (1986). Vouchers were deposited in the collection de Mirmecologia do Alto Tietê. Samples were kept in ethanol and stored at −20°C until DNA extraction was performed.

### DNA extraction and bacterial DNA sequencing

Whole ant workers were used for DNA extraction using DNeasy PowerSoil Kit (Qiagen, Germantown, MD USA). Methods from Moreau (2014) were used to avoid contamination. To remove contaminants that may have been added in the process of obtaining the data, three negative controls were added to extraction methods as well as subsequent 16S rRNA amplifications and library sequencing. The negative controls correspond to water instead of DNA during the polymerase chain reaction (PCR). Following Earth Microbiome Project (EMP) protocols (https://earthmicrobiome.org/) also described in Caporaso et al. (2012), the V4 region of 16S rRNA was amplified (515F/806R primers) in three samples. PCR reactions followed recommendations from the Taq Platinum Kit (Invitrogen, Schwerte, Germany) with 2.5 µL Buffer 10X, 1.0 µL MgCl_2_ 50 mM, 1.0 µL dNTPs 10 mM, 1.0 µL bovine serum albumin (BSA) 1 mg/mL, 0.5 µL 515 F primer 10 µM, 0.5 µL 806R primer 10 µM, 0.5 µL Taq Platinum 5U/µL, 5 µL template DNA (10 ng/µL), and 13.0 µL PCRWater (Certified DNA-free). The reaction was submitted to the thermal cycler under the following conditions: 95 °C for 3 min, 30 cycles at 95 °C for 30 s, 60 °C for 30 s, and 72 °C for 30 s, with final of 72 °C for 10 min and 4 °C hold. Purification was performed with the Ampure X beads and adapter connections (NEXTERA XT) were also performed following the manufacturer’s recommendations. The samples were quantified by Nanodrop (Thermo Fisher Scientific, Waltham, MA, USA). Pooling and normalization were performed so that samples had the same concentration. Then, the DNA pool was diluted down to 2 nM, denatured, and diluted to a final concentration of 16 pM with a 20% PhiX for sequencing on the Illumina MiSeq with run 2 × 250 pb at the Centro de Genômica (ESALQ, Piracicaba, SP, Brazil).

### Bioinformatic analysis

The Qiime2 platform (Bolyen et al. 2019) was used to process paired-end demultiplexed fastq files following the Casava 1.8 protocol. Quality control and feature table construction were performed using the DADA2 plugin, which ensured more accurate results (McDonald et al. 2012; Callahan et al. 2016). Paired-end reads were trimmed, and taxonomic identification of amplicon sequence variants (ASVs) was achieved using the SILVA_132_QIIME database, based on 99% similarity (Quast et al. 2013; Yilmaz et al. 2014; Callahan et al. 2016). SILVA Tree Viewer (Beccati et al. 2017) was used to confirm taxonomic identification of amplicon sequence variants. This study uses taxonomic identification to an order level. In results where order level ASV identification was not possible, the class of bacteria was reported.

To eliminate potential contaminants, including mitochondrial and chloroplast sequences, negative controls were applied through the prevalence method in the Decontam (Davis et al. 2018) in R (R Development Core Team 2023). A phylogenetic tree of the remaining ASVs was then constructed using the “align-to-tree-mafft-fasttree” command (Katoh and Standley 2013). This phylogeny was created prior to performing diversity analyses. For these analyses, samples were normalized to a sequencing depth of 3400 reads to ensure all samples in the dataset were retained (Price et al. 2010; Vázquez-Baeza et al. 2013).

To analyze phylogenetic variance, Permutational Multivariate Analysis of Variance (PERMANOVA) was conducted in a “pairwise” manner using UniFrac distance metrics. The UniFrac method provided a robust non-parametric approach to assessing multivariate datasets measuring the association of bacterial community composition between samples (Anderson 2001). To ensure homogeneity of multivariate dispersion among samples, Permutational Analyses of Multivariate Dispersions (PERMDISP) (Anderson et al. 2006) were conducted using Qiime2 qiime diversity permdisp function for all treatment groups using UniFrac distance metrics. To determine if “core” bacteria, (bacteria found in > 50% of samples) were present within the bacterial communities of samples, qiime feature table core-features plugin (Bolyen et al. 2019) was used for analyses.

SIMPER (Similarity Percentage) multivariate analyses, using the Bray-Curtis index in Past4, were conducted to further confirm ASVs’ roles in shaping the bacterial communities (Clarke 1993; Hammer et al. 2001; McCune and Grace 2002). Principal coordinates analysis (PCoA) and network analyses of the bacterial communities were performed using the phyloseq (McMurdie and Holmes 2013) and ggplot2 (Wickham, 2009) packages in R. Network analyses utilized a Bray-Curtis index to quantify pairwise differences in the abundance of features to calculate similarity of bacterial community composition. Network analyses were performed using the tidyverse (Wickham et al. 2019), readxl (Wickham and Bryan 2025), phyloseq (McMurdie and Holmes 2013), readr (Wickham et al. 2023), tibble (Müller and Wickham 2025), and ggplot2 (Wickham, 2009) packages in R. All raw sequence data generated in this study are publicly accessible at the National Center for Biotechnology Information (NCBI), under SRA accession number PRJNA556881 and study SAMN12373152.

## Results

### Bacterial community composition in Atta species

**Fig. 1:**
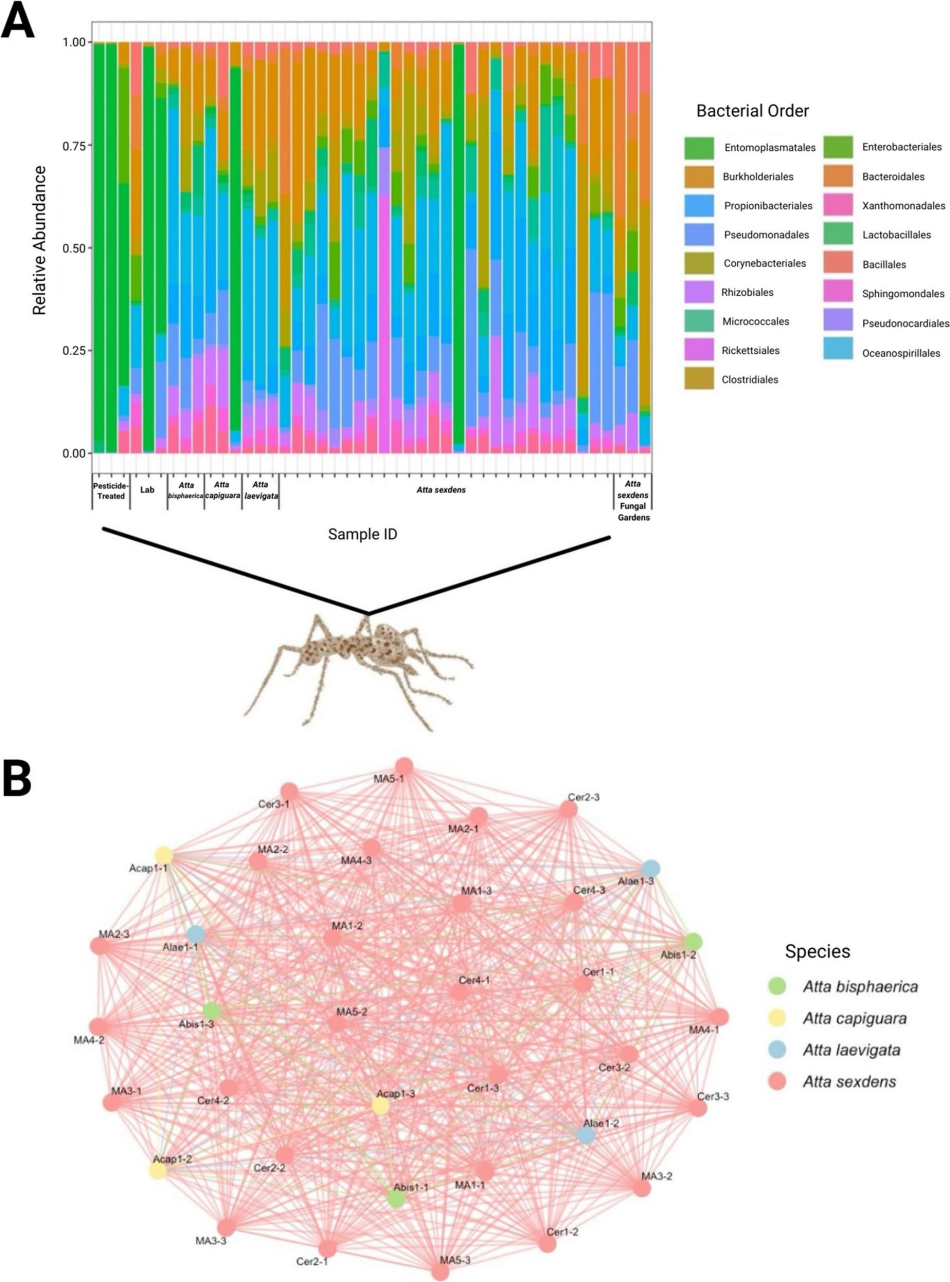
Bacterial community composition and compositional similarity among *Atta* samples. **A** Barplot of relative abundance of dominant bacterial orders across all four *Atta* species (*Atta laevigata*, *Atta bisphaerica*, *Atta capiguara*, *Atta sexdens*). The most abundant bacterial orders found among *Atta* species were Entomoplasmatales (10.6%), Burkholderiales (9.7%), and Propionibacteriales (9.3%) followed by others in smaller quantities. The legend is arranged from most to least abundant taxa. **B** Bray-Curtis-based similarity network illustrating pairwise compositional similarity among samples. Each node represents a sample, and node color indicates species identity

Each sample was rarefied to a depth of 3400 reads; no samples were excluded due to this cutoff, and 39 samples remained. Sampling ranged from a maximum of 182,624 reads to a minimum of 7539 reads. Bioinformatic analysis of bacterial communities was performed at an order taxonomic level. All samples were analyzed for “core” bacteria (defined as bacterial taxa present in > 50% of samples); no core bacterial orders were identified across all samples. Across all four *Atta* species (*Atta laevigata*, *Atta bisphaerica*, *Atta capiguara*, *Atta sexdens*), the most abundant bacterial orders were Entomoplasmatales (10.6%), Burkholderiales (9.7%), and Propionibacteriales (9.3%), followed by others in lower relative abundances (Fig. 1A). Samples of *Atta sexdens* fungal gardens were analyzed in addition to entire pesticide-treated *Atta sexdens* workers, and lab-reared *Atta sexdens* (Nogueira et al. 2022). The most abundant bacterial orders overall among samples of *Atta sexdens* fungal gardens were found to be Clostridiales (33%), Bacteroidales (23%), and Bacillales (11%), followed by others in smaller quantities. Lab-reared *Atta sexdens* were dominated by Entomoplasmatales (46%), Burkholderiales (11%), and Bacteroidales (6%), followed by others in lower relative abundances. Pesticide-treated *Atta sexdens* were primarily composed of Entomoplasmatales (75%), Enterobacteriales (13%), and Xanthomonadales (3%), followed by others in lower relative abundances. Visualization of relative bacterial abundance for each sample is shown in Fig. 1A. Figure 1B presents a Bray-Curtis based similarity network illustrating pairwise compositional similarity among samples.

### Beta diversity

Beta diversity determined using established beta diversity Weighted Unifrac and Unweighted Unifrac metrics (Anderson 2001; Lozupone and Knight 2005; Lozupone et al. 2006, 2011; Chen et al. 2012) provided insight into the composition and diversity of bacterial communities between samples. These results were then analyzed with Compositional Pairwise Permutational Multivariate Analysis of Variance (PERMANOVA) tests to determine whether the pairwise composition of the bacterial communities between samples of species compared had more similar or dissimilar bacterial communities (Lozupone et al. 2011).

A significant difference was detected in the composition (PERMANOVA Unifrac unweighted, *P*-value = 0.029, Pseudo *F* = 1.159) as well as in the abundance (PERMANOVA Unifrac weighted, *P*-value = 0.036, Pseudo *F* = 1.046) of bacterial communities among different *Atta* species. To verify that PERMANOVA results reflected shifts in group centroids rather than unequal variability between groups, a PERMDISP was conducted (*P*-value = 0.606), confirming that significant differences were not a result of heterogeneity of difference.

A compositional pairwise analysis was conducted to compare further, revealing that this difference was driven by the *Atta laevigata* and *Atta sexdens* species (PERMANOVA Unifrac unweighted: *P*-value = 0.001 and Pseudo *F* = 2.07). SIMPER analysis revealed that Burkholderiales (more abundant in *Atta laevigata*) is driving the most difference between *A. laevigata* and *A. sexdens*, contributing to 10.9% of the difference, followed by Corynebacteriales (more abundant in *Atta laevigata*) with 8.0%, Propionibacteriales (more abundant in *Atta sexdens)* with 6.2%, followed by others in less abundance (Table 1).

**Table 1:** Similarity percentage (SIMPER) of bacterial communities driving the difference between *Atta sexdens* vs. *Atta laevigata*. SIMPER multivariate analysis (using Bray-Curtis index) conducted of main bacteria driving significant compositional difference between *Atta sexdens* and *Atta laevigata.* SIMPER indicates specific amplicon sequence variants (ASVs) to bacterial community makeup between *Atta sexdens* and *Atta laevigata* samples. SIMPER stipulates that Burkholderiales contribute most to the difference (10.9% contribution) among species, with higher abundance in *Atta laevigata* samples. Results reported at an order taxonomic level. If unavailable, class taxonomic level was reported

Species	Average dissimilarity (%)	Most influential ASV/taxonomy	Percent contribution to difference	Cumulative (%)	Samples with higher ASV abundance
*Atta sexdens* vs. *Atta laevigata*	7.29	Burkholderiales	10.93	10.93	*A. laevigata*
	5.33	Corynebacteriales	8.00	18.92	*A. laevigata*
	4.11	Propionibacteriales	6.16	25.08	*A. sexdens*
	2.79	Entomoplasmatales	4.18	29.26	*A. sexdens*
	2.45	Gammaproteobacteria	3.68	36.95	*A. laevigata*
	2.17	Pseudomonadales	3.26	40.21	*A. sexdens*
	2.06	Micrococcales	3.09	43.30	*A. sexdens*
	1.98	Lactobacillales	2.97	46.26	*A. laevigata*
	1.84	Rhizobiales	2.76	49.02	*A. sexdens*
	1.83	Alphaproteobacteria	2.75	51.76	*A. laevigata*
	1.80	Clostridiales	2.69	54.46	*A. sexdens*
	1.53	Bacteroidales	2.29	56.74	*A. sexdens*
	1.43	Enterobacteriales	2.15	58.89	*A. sexdens*
	1.34	Sphingomondales	2.00	60.90	*A. laevigata*
	1.05	Bacillales	1.57	62.47	*A. laevigata*
	1.04	Chthoniobacterales	1.56	64.03	*A. sexdens*

Next, the bacterial communities of *Atta sexdens* collected in their natural habitat were compared with those of lab-reared *Atta sexdens* samples. Both compositional (PERMANOVA Unifrac unweighted, *P*-value = 0.001, Pseudo *F* = 2.02) and abundance (PERMANOVA Unifrac weighted, *P*-value = 0.007, Pseudo *F* = 3.611) revealed significant differences between the bacterial communities of natural habitat and lab *Atta sexdens.* To verify that PERMANOVA results reflected shifts in group centroids rather than unequal variability between groups, a PERMDISP was conducted (*P*-value = 0.352), confirming that significant differences were not a result of heterogeneity of difference.

SIMPER analysis revealed the bacteria Entomoplasmatales (more abundant in natural habitat *A. sexdens*) was driving the most difference between natural habitat and lab samples, contributing 24.1%, followed by Burkholderiales (more abundant in natural habitat samples) with 7.9%, followed by Propionibacteriales (more abundant in natural habitat samples) with 6.6%, and others in less abundance (Table 2).

**Table 2:** Similarity percentage (SIMPER) of bacterial communities driving the difference between natural habitat and lab *Atta sexdens.* SIMPER multivariate analysis (using Bray-Curtis index) was conducted to reveal main bacteria driving significant compositional difference between natural habitat and lab *Atta sexdens* samples. SIMPER indicates specific amplicon sequence variants (ASVs) to bacterial community makeup between natural habitat and lab *Atta sexdens*. SIMPER stipulates that Entomoplasmatales contribute most to the difference (24.1% contribution) among samples, with higher abundance in natural habitat *Atta sexdens* samples. Results are reported at an order taxonomic level. If unavailable, class taxonomic level was reported

Species	Average dissimilarity (%)	Most influential ASV/taxonomy	Percent contribution to difference	Cumulative (%)	Samples with higher ASV abundance
Natural habitat vs. lab *Atta sexdens*	20.89	Entomoplasmatales	24.06	24.06	Natural habitat
	6.85	Burkholderiales	7.89	31.95	Natural habitat
	5.75	Propionibacteriales	6.62	38.58	Natural habitat
	4.24	Pseudomonadales	4.89	43.46	Natural habitat
	3.54	Bacteroidales	4.07	47.54	Lab
	2.96	Gammaproteobacteria	3.41	50.95	Natural habitat
	2.94	Clostridiales	3.39	54.34	Natural habitat
	2.94	Corynebacteriales	3.38	57.72	Natural habitat
	2.94	Enterobacteriales	3.38	61.10	Lab
	2.92	Alphaproteobacteria	3.36	64.46	Natural habitat
	2.66	Bacillales	3.07	67.53	Lab
	2.44	Micrococcales	2.81	70.34	Natural habitat
	2.35	Rhizobiales	2.70	73.05	Natural habitat
	1.82	Rickettsiales	2.10	77.67	Natural habitat
	1.81	Xanthomonadales	2.08	79.75	Natural habitat

**Fig. 2:**
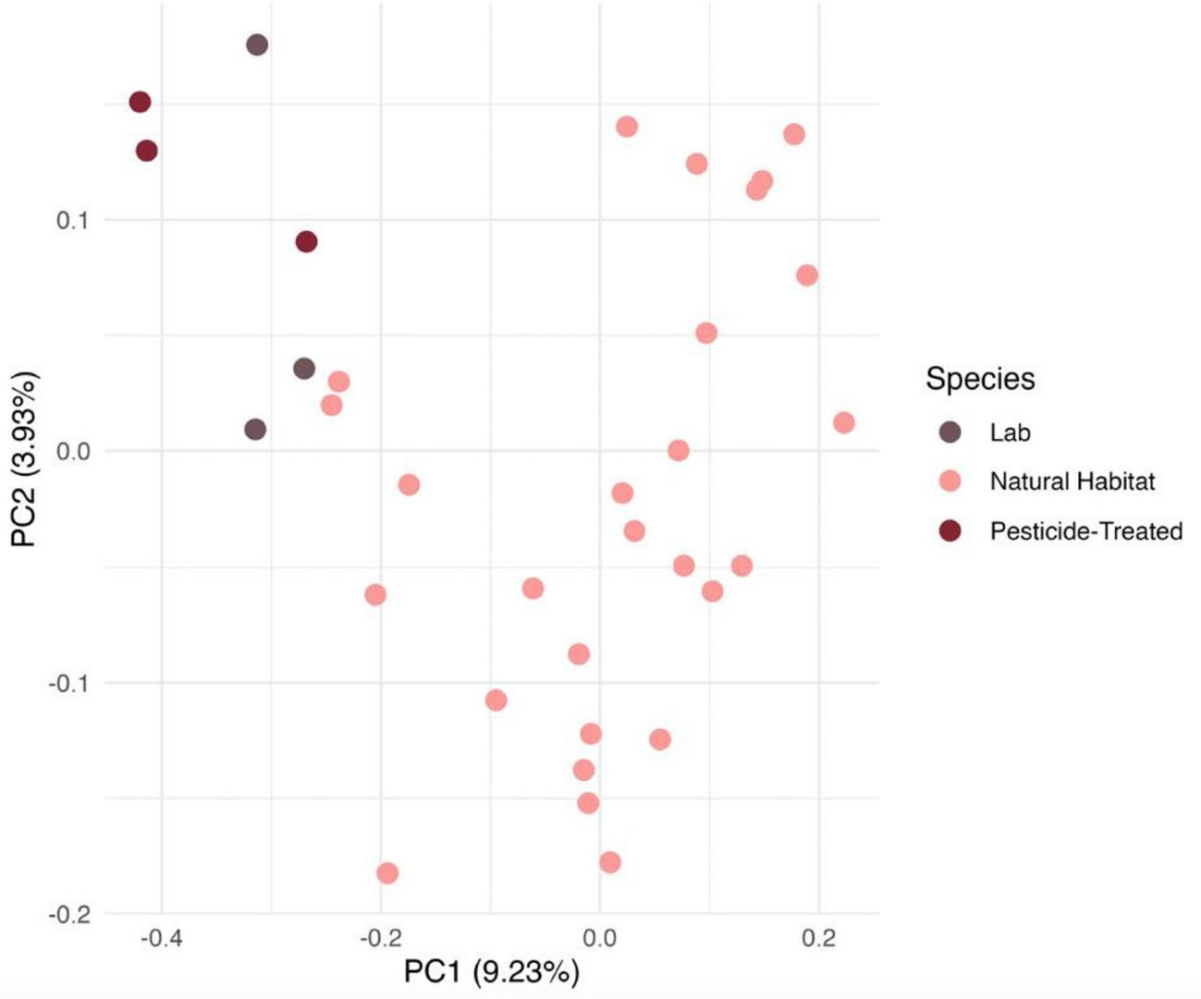
Unifrac unweighted principal coordinate analysis (PCoA) of lab, natural habitat, and pesticide-treated *Atta sexdens.* Each dot represents the bacterial community composition of one sample. The closer the dots are together, the more similar their bacterial community composition. No significant difference found between the bacterial community composition of lab and pesticide-treated *Atta sexdens* samples (PERMANOVA Unifrac unweighted: *P*-value = 0.496 and Pseudo *F* = 1.09, Fig. 2)

In addition to the 27 samples of *A. sexdens* workers collected in their natural habitat and three healthy *A. sexdens* kept in the lab, we treated three additional lab-reared *A. sexdens* with pesticide and compared bacterial communities of all three sample types. A significant difference was found overall among natural habitat, lab, and pesticide-treated *A. sexdens* samples. To compare natural habitat and pesticide-treated *A. sexdens*, a pairwise permutational multivariate analysis of variance (PERMANOVA) was conducted. Significant difference was found with an overall composition unweighted *P*-value = 0.001, Pseudo *F* = 2.54 and an abundance-weighted *P*-value = 0.002, Pseudo *F* = 7.868. To verify that PERMANOVA results reflected shifts in group centroids rather than unequal variability between groups, a PERMDISP was conducted (*P*-value = 0.967), confirming that significant differences were not a result of heterogeneity of difference. There was no significant difference found between the bacterial communities of lab and pesticide-treated *A. sexdens* samples (PERMANOVA Unifrac unweighted: *P*-value = 0.496 and Pseudo *F* = 1.09, Fig. 2).

SIMPER analysis between the bacterial communities of natural habitat and pesticide-treated *Atta sexdens* samples showed that Entomoplasmatales (more abundant in pesticide-treated samples) drives the most difference, contributing 50.6%, followed by Enterobacteriales (more abundant in natural habitat samples) with 7.4%, followed by Propionibacteriales (more abundant in natural habitat samples) with 4.4%, and others in less abundance (Table 3).

**Table 3:** Similarity percentage (SIMPER) of bacterial communities driving the difference between natural habitat and pesticide-treated *Atta sexdens.* SIMPER multivariate analysis (using Bray-Curtis index) was conducted to reveal main bacteria driving significant compositional difference between natural habitat and pesticide-treated *Atta sexdens.* SIMPER indicates specific amplicon sequence variants (ASVs) to bacterial community makeup between natural habitat and pesticide-treated *Atta sexdens*. SIMPER stipulates that Entomoplasmatales contributes most to the difference (55.6% contribution) among samples, with higher abundance in natural habitat *Atta sexdens* samples. Results reported at an order taxonomic level. If unavailable, class taxonomic level was reported

Species	Average dissimilarity (%)	Most influential ASV/taxonomy	Percent contribution to difference	Cumulative (%)	Samples with higher ASV abundance
Natural habitat vs. pesticide-treated *Atta sexdens*	46.50	Entomoplasmatales	50.55	50.55	Pesticide treated
	6.81	Enterobacteriales	7.40	57.95	Natural habitat
	4.09	Propionibacteriales	4.44	62.39	Natural habitat
	3.24	Corynebacteriales	3.52	65.91	Natural habitat
	3.02	Burkholderiales	3.28	69.2	Natural habitat
	2.29	Pseudomonadales	2.49	71.68	Natural habitat
	2.01	Micrococcales	2.19	73.87	Natural habitat
	1.95	Lactobacillales	2.12	75.99	Natural habitat
	1.62	Clostridiales	1.76	77.75	Natural habitat
	1.61	Xanthomonadales	1.75	79.50	Pesticide treated
	1.51	Rhizobiales	1.64	81.14	Pesticide treated
	1.44	Gammaproteobacteria	1.57	82.71	Natural habitat
	1.39	Alphaproteobacteria	1.51	84.22	Natural habitat
	0.92	Bacteroidales	1.00	86.49	Natural habitat
	0.85	Sphingomondales	0.93	87.42	Natural habitat

Samples of *Atta sexdens* fungal gardens were compared with all species (*Atta laevigata*, *Atta bisphaerica*, *Atta capiguara*, *Atta sexdens*), including lab, natural habitat, and pesticide-treated *Atta sexdens.* To compare the bacterial communities of *Atta sexdens* fungal gardens and all four leaf-cutter species (including separate analyses for each *A. sexdens* condition), a pairwise permutational multivariate analysis of variance (PERMANOVA) was conducted. There was found to be a significant difference in the composition (PERMANOVA Unifrac unweighted, *P*-value = 0.001, Pseudo *F* = 2.17) and abundance (PERMANOVA Unifrac weighted, *P*-value = 0.003, Pseudo *F* = 3.306) between the bacterial communities of natural habitat *Atta sexdens* and *Atta sexdens* fungal garden samples. To verify that PERMANOVA results reflected shifts in group centroids rather than unequal variability between groups, a PERMDISP was conducted (*P*-value = 0.907), confirming that significant differences were not a result of heterogeneity of difference.

A SIMPER analysis revealed that Clostridiales (contributing 13.1%, more abundant in fungal gardens) was driving the most difference between *Atta sexdens* fungal gardens and natural habitat *Atta sexdens*, followed by Bacteroidales (contributing 11.0%, more abundant in fungal gardens), Propionibacteriales (contributing 7.2%, more abundant in natural habitat *Atta sexdens*), and others in less abundance (Table 4).

**Table 4:** Similarity percentage (SIMPER) of bacterial communities driving the difference between *Atta sexdens* fungal gardens and natural habitat *Atta sexdens.* SIMPER multivariate analysis (using Bray-Curtis index) shows main bacteria driving significant compositional difference between *Atta sexdens* fungal gardens and natural habitat *Atta sexdens*. SIMPER indicates specific amplicon sequence variants (ASVs) to bacterial community makeup between fungal garden samples and natural habitat *Atta sexdens* samples. SIMPER stipulates that Clostridiales contribute most to the difference (13.1% contribution, more common in fungal gardens) and others in less abundance. Results reported at an order taxonomic level. If unavailable, class taxonomic level was reported

Species	Average dissimilarity (%)	Most influential ASV/taxonomy	Percent contribution to difference	Cumulative (%)	Samples with higher ASV abundance
*Atta sexdens* vs. fungal gardens	10.32	Clostridiales	13.12	13.12	Fungal gardens
	8.63	Bacteroidales	10.97	24.09	Fungal gardens
	5.67	Propionibacteriales	7.21	31.30	*Atta sexdens*
	4.35	Corynebacteriales	5.53	36.83	*Atta sexdens*
	3.96	Bacillales	5.03	41.86	Fungal gardens
	3.60	Burkholderiales	4.57	46.43	*Atta sexdens*
	3.39	Pseudomonadales	4.31	50.74	*Atta sexdens*
	3.28	Entomoplasmatales	4.17	54.91	*Atta sexdens*
	2.86	Lactobacillales	3.64	58.55	*Atta sexdens*
	2.74	Micrococcales	3.48	62.03	*Atta sexdens*
	2.38	Enterobacteriales	3.03	65.06	*Atta sexdens*
	2.10	Rhizobiales	2.67	67.73	*Atta sexdens*
	2.07	Alphaproteobacteria	2.63	70.36	*Atta sexdens*
	2.02	Gammaproteobacteria	2.57	72.93	*Atta sexdens*
	1.87	Selenomonadales	2.37	77.72	Fungal gardens
	1.12	Xanthomonadales	1.42	79.14	*Atta sexdens*

## Discussion

The *Atta* genus of ants is fungus-cultivating (tribe Attini) and leaf-cutting, demonstrating a complex symbiosis with their environments (Sapountzis et al. 2019). Several factors of influence have been established to be responsible for the diversity of attine bacterial communities, including mutualistic function (Andersen et al. 2013), phylogeny (Sapountzis et al. 2019), diet (Chanson et al. 2023), development stage (Zhukova et al. 2017), and caste (Vieira et al. 2017). This study found the bacterial communities of allopatric *Atta* spp. to be significantly different, driven by a significant difference between the bacterial communities of *A. sexdens* and *A. laevigata* in composition (PERMANOVA Unifrac unweighted, *P*-value = 0.001, Pseudo *F* = 2.02) and abundance (PERMANOVA Unifrac weighted, *P*-value = 0.007, Pseudo *F* = 3.611). *Atta sexdens* reared in the lab were found to have significantly different bacterial communities from those that were reared in their natural habitat in composition (PERMANOVA Unifrac unweighted, *P*-value = 0.001, Pseudo *F* = 2.02) and abundance (PERMANOVA Unifrac weighted, *P*-value = 0.007, Pseudo *F* = 3.611). Bacterial communities of pesticide-treated *A. sexdens* significantly differed in composition to natural habitat *A. sexdens* (PERMANOVA Unifrac unweighted *P*-value = 0.001, Pseudo *F* = 2.54) and abundance (PERMANOVA Unifrac weighted *P*-value = 0.002, Pseudo *F* = 7.868). Bacterial communities of pesticide-treated *A. sexdens*, collected from lab-reared colonies, did not significantly differ from *A. sexden*s kept in the laboratory. Bacterial communities of mutualistic *A. sexden*s fungal garden samples were found to significantly differ in composition (PERMANOVA Unifrac unweighted, *P*-value = 0.001, Pseudo *F* = 2.17) and abundance (PERMANOVA Unifrac weighted, *P*-value = 0.003, Pseudo *F* = 3.306) from the bacterial communities of the symbiont, *A. sexdens.*

It is important to note that this study identifies bacteria present to an order level. Any functional roles of each taxon discussed should be considered indicative rather than definitive, given that this taxonomic rank encompasses considerable ecological diversity. Discussion of each taxon is intended to contextualize our findings within the existing literature and to highlight studies that may help guide future inquiry. The primary aim of this work is to draw attention to the significant differences detected among the bacterial communities of *Atta* species and to provide a foundation for subsequent research on their variable bacterial communities. These findings should be interpreted as preliminary, and further investigation with expanded sample sizes will be essential.

When analyzing the bacterial community composition of *Atta* collected in their natural habitats, several taxa were identified across species. Entomoplasmatales (genus *Mesoplasma*) was the most common ASV, with a 10.6% relative frequency in natural samples. High abundance of Entomoplasmatales has been found in the postpharyngeal gland (PPG) of *A. sexdens rubropilosa* queens. Vieira et al. (2017) suggest that this may come from a potential role of Entomoplasmatales in lipid nutrition, as the PPG has an important role in lipid absorption, metabolization, and mobilization (Vieira et al. 2015, 2017). Entomoplasmatales have also been found in the fat body cells of *Acromyrmex* ants, a genus of leaf-cutting fungus-cultivating ants, across different colonies (Sapountzis et al. 2015). Sapountzis et al. (2018) suggest that the role of Entomoplasmatales in *Atta* may instead lie in augmenting the innate chitin processing ability in *Atta* (Lehane 1997; Zhao et al. 2019). *Atta sexdens* specifically possess chitinase AschtII-C4B1, recently suggested to be inherited from a mutualistic relationship with fungi (Micocci et al. 2023). Interestingly, Meirelles et al. (2016a, b) found a high concentration of Entomoplasmatales in fungal pellets carried by foundress *Atta* queens. Entomoplasmatales has also been detected in *A. sexdens* (Ramalho et al. 2020a, b), but their causal relationship with *Atta* species and their symbiotic fungus remains unclear and warrants further investigation.

Burkholderiales has been found to be significantly abundant in the guts and whole workers of herbivorous *Cephalotes* ants (Russell et al. 2009; Anderson et al. 2012). In herbivorous species, Burkholderiales has been linked to nitrogen metabolism (Russell et al. 2009; Hu et al. 2018), playing a role in the recycling of uric acid to synthesize essential amino acids and aid in the breakdown of urea in *Cephalotes* (Hu et al. 2018). Burkholderiales has also been found to be involved in the nitrogen metabolization of herbivorous *Tetraponera* ants (Van Borm et al. 2002). Burkholderiales has been found in high abundance in *A. sexdens rubropilosa* whole workers and queens (Vieira et al. 2017) as well as in the abdomens of *A. sexdens* and *A. laevigata*, suggested to be involved in nitrogen fixing (Zani et al. 2021). Burkholderiales has also been found in high abundance in other fungivorous ants, *Trachymyrmex* (Allert 2017). While this bacterium may contribute to nitrogen fixing in *Atta* species, nitrogen fixing has been strongly associated with bacteria of the Enterobacteriales order from fungal gardens of *Atta* (Pinto-Tomás et al. 2009). Nitrogen fixing may not be the primary function of these bacteria in *Atta* as Burkholderiales has also been found to produce a wide spectrum of antibiotics effective against *Aspergillus* fungi spp., an entomopathogenic fungi found to be lethal in *A. bisphaerica* (Anderson et al. 2012; Ribeiro et al. 2012).

Propionibacteriales is the next most abundant order found among *Atta* collected in natural habitats. This order has not yet been documented to be found in ants. *Propionibacterium*, a genus of Propionibacteriales, has been found in fragments of the fungal gardens of lower attines, *Mycoceprus smithii* in Panama, but not in the bacterial community of these ants. Propionibacteriales has been reported as abundant in continuously cropped soils, serving as a biomarker of cropped soils (Ma et al. 2023). Strains of Propionibacteriales are commonly found in mammal gastrointestinal microbiota and are used to industrially produce propionic acid (Plé et al. 2016; Grinstead and Barefoot., 1992). Future work should examine the interaction of these bacteria and ants’ hosts.

### Difference in bacterial communities among Atta species

Bacterial communities of all four native species of *Atta* from São Paulo collected in their natural habitat were compared, revealing a significant difference between *A. sexdens* and *A. laevigata* specifically*.* The three main bacteria driving this difference were Burkholderiales, Corynebacteriales, and Propionibacteriales, each present in both species compared (Table 1). Burkholderiales and Corynebacteriales were found to be more abundant in *A. laevigata.* It is important to note that while results are significant, a limitation of this study is the smaller sample sizes of species, especially *A. laevigata*, *A. capiguara*, and *A. bisphaerica* species*.* We suggest future studies with larger sample sizes of each species be conducted to verify significance. Colony variability should also be explored.

Corynebacteriales is less susceptible to many antibiotics, attributed to their unique cell wall permeability (Bernard 2012). It has been present as a top bacterium in abundance across all eight different subfamilies of ants, including Myrmicinae, housing *Atta* (Chanson et al. 2023). Corynebacteriales has also been found in high abundance on the exoskeleton and associated fungi of *Cyphomyrex minuts* ants (Medina-Rivera 2012). Nepel et al. (2023) note Corynebacteriales presence in *Azteca* nests with decreasing abundance proportional to colony development.

Propionibacteriales was the third most prevalent, driving the difference between *A. sexdens* and *A. laevigata*, being more abundant in *A. sexdens.* Although the cause of the presence in ants is unknown, there is long-term presence in inflammatory gut microbiota in mammals (Grinstead and Barefoot 1992; Plé et al. 2016; Laue et al. 2019).

### Difference in bacterial communities between natural habitat and lab Atta sexdens

The bacterial communities of *Atta sexdens* samples kept in the lab were found to be significantly different from the bacterial communities of all *Atta* spp. collected in their natural habitat. The top three orders of bacteria driving this difference were Entomoplasmatales, Burkholderiales, and Propionibacteriales (Table 2). The association of these orders with leaf-cutter ants has already been previously discussed in this study.

It has been well established that collection sites play a substantial role in the composition of the microbiota of other organisms (Cox and Gilmore 2007; Chandler et al. 2011; Staubach et al. 2013; Didion et al. 2022). Despite this, this study is the first to explore the difference in bacterial communities from natural habitats versus laboratory conditions of *Atta.* Considering that lab colonies of ants are common practice to answer several myrmecological questions, it is important to explore whether collecting in the lab will affect the microbiota, therefore skewing results and misrepresenting bacterial communities. While collection sites may not provide a significant difference in genus of ants with simple or well-established core bacterial communities, it is crucial for genera of ants with diverse and transient bacterial communities (Ramalho et al. 2020a, b).

### Difference in bacterial communities between natural habitat, lab, and pesticide-treated Atta sexdens

There are no studies to date that analyze for significant difference between the bacterial communities of laboratory reared ants and ants collected in their natural environments. There have been studies looking at bacterial community differences between *Camponotus* ants collected in their natural habitats and reared in the lab (He et al. 2014). Different bacteria found were reported, but significant compositional difference in bacterial communities was not tested for. There have, however, been studies conducted in other insects such as *Drosophila* (Staubach et al. 2013) and *Culicidae* (Didion et al. 2022), finding significant compositional differences in the bacterial communities of samples reared in the lab versus collection in their natural environment. The rearing of samples in laboratory conditions has been found to significantly affect the bacterial communities of *Atta* in this study. This study takes pesticide treatment into account by separate pairwise comparison of natural habitat collected and lab *Atta sexdens*. Sulfuramid was used for pesticide treatment of ants in this study. Sulfuramid is a commonly used pesticide against *Atta* despite extreme hazard to humans and other organisms (Della Lucia et al., 2020; Bejarano et al. 2019). Sulfuramid (a perfluoroalkyl compound) is a top chemical class found to produce the most oxidative stress via lipid peroxidation, causing cell loss (Slotkin and Seidler 2010).

The most abundant bacterial orders found in the composition of pesticide-treated *Atta sexdens* were Entomoplasmatales, Enterobacteriales, and Xanthomonadales. Entomoplasmatales presence is substantial, accounting for 75% of the bacterial composition of pesticide-treated *Atta sexdens* samples. Note that Entomoplasmatales was also the main bacterial order among healthy *Atta* spp., suggesting that this bacteria could potentially be conserved. The next most abundant order in pesticide-treated *Atta sexdens* is Enterobacteriales. This bacterial order is known to benefit B-vitamin synthesis, sulfate nitrate reduction, nitrogen recycling, amino acid production, and key aspects of sulfur metabolism (Béchade et al. 2021). The presence of Enterobacteriales has been found in other ant-cultivated fungi, with a high proportion found in the associated fungi of *Trachymyrmex* ants (Allert 2017). It is unclear why there is an abundance of Enterobacteriales in the bacterial composition of pesticide-treated *Atta sexdens* in this study; further research is needed to resolve this. Xanthomonadales was the third most abundant order found in pesticide-treated *Atta.* The function of this bacterial order in *Atta i*s unclear. It has been found to be abundant in the guts of herbivorous ants (Russell et al. 2009; Kautz et al. 2013; Chanson et al. 2021, 2023; Flynn et al. 2021), notably abundant in *Cephalotes* bacterial communities. Xanthomonadales has also been found to be highly abundant in lower attine workers, fungal gardens, and soil (Kellner et al. 2015). Xanthomonadales is broadly categorized alongside four other bacterial orders claimed to play a role in amino acid synthesis and nitrogen recycling (Chanson et al. 2023), but this does not encompass specificity of Xanthomonadales function. More studies need to be conducted to further explore the origin and function of this order in *Atta*.

To identify how pesticide treatment and extensive hazards affected the bacterial communities of *Atta*, the bacterial communities of pesticide-treated *Atta sexdens* were compared to the bacterial communities of natural habitat *Atta sexdens*.

Entomoplasmatales was the driving factor behind the difference between pesticide-treated and natural habitat *A. sexdens*, more abundant in pesticide-treated *A. sexdens*. Enterobacteriales drove the second most amount of difference between bacterial communities of pesticide-treated and natural habitat *A. sexdens*, much more abundant in pesticide-treated *Atta* (Table 3). Propionibacteriales drove the third most amount of difference between bacterial communities, highly abundant in natural habitat *A. sexdens*, and minimally present in pesticide-treated *A. sexdens*. Shockingly, the bacterial community of lab *A. sexdens* was not significantly different from the bacterial community of pesticide-treated *A. sexdens*. Perhaps future studies with other pesticide concentrations and other treatment timespans may show different results. Additionally, it is important to note that the pesticide-treated samples were reared in the lab prior to treatment. While there is still significant difference between all three sample groups that were lab reared, pesticide treated, and collected in their natural environment, the act of removing the ants from their natural environment may be significantly influencing the change in bacterial communities in potential addition to pesticide treatment. This emphasizes the significance of the effect of collection conditions when studying *Atta* and calls for future research to compare bacterial communities of pesticide-treated ants in and out of the lab.

### Difference in bacterial communities between fungal gardens and all Atta species

The bacterial communities of *Leucoagaricus gongylophorus* were compared to the bacterial community composition of their coevolutionary symbiont, *Atta*. This study seeks to identify bacterial diversity of the fungi and compare it to that of *Atta* spp. The most compositionally abundant bacterial orders found in the fungi were Clostridiales, Bacteroidales, and Bacillales.

**Fig. 3:**
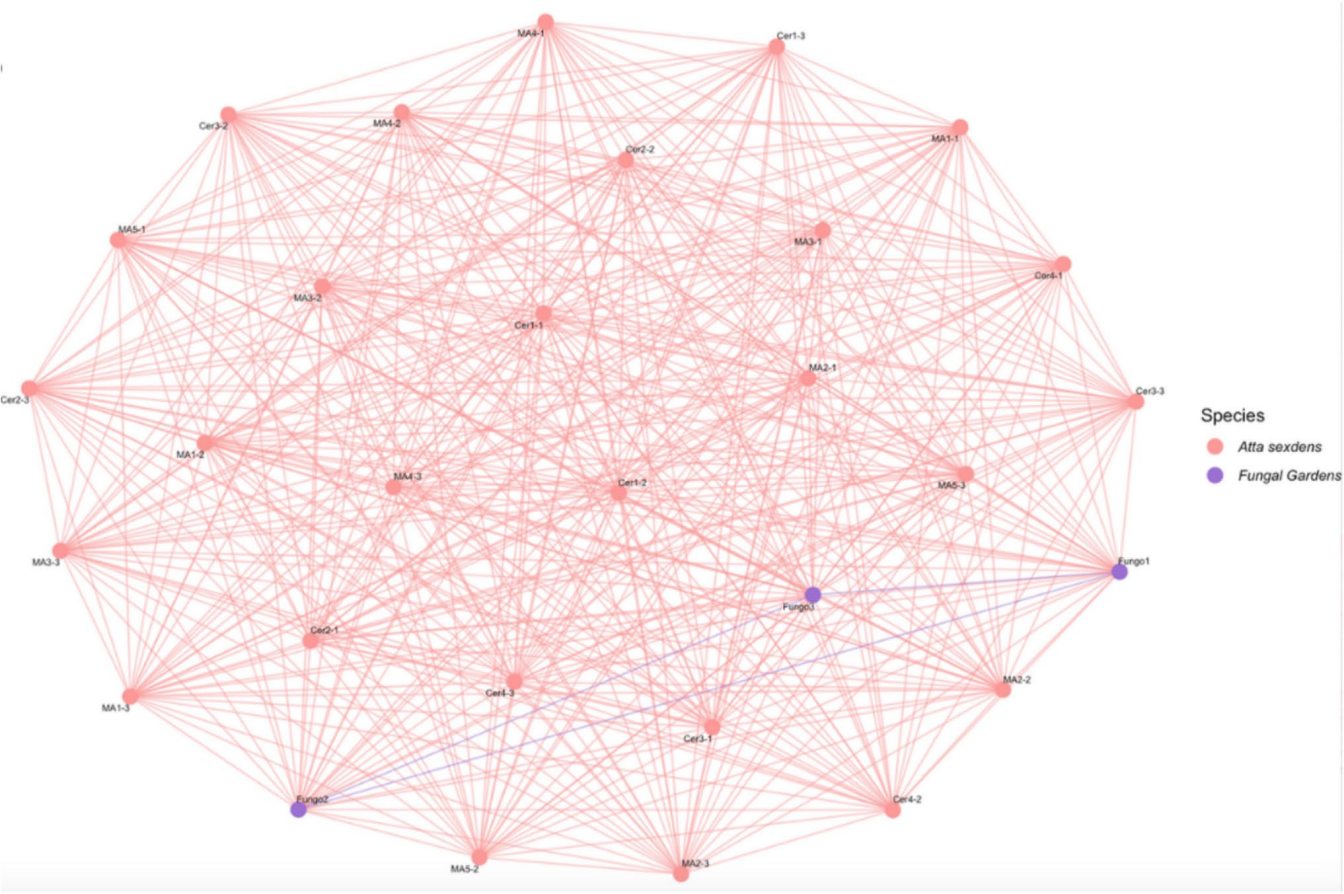
Network analysis showing Bray-Curtis-based similarity network illustrating pairwise compositional similarity among *Atta sexdens* fungal gardens and natural habitat *Atta sexdens* samples. Network analysis analyzes ecological similarity based on relatedness of bacterial community composition among *Atta sexdens* fungal gardens and natural habitat *Atta sexdens* samples included in this study. Each red node represents one sample of natural habitat *Atta sexdens*, and each purple node represents one sample of *Atta sexdens* fungal garden. The line between each node represents one bacterial order shared between the nodes, or samples, connected

A significant difference was found between natural habitat *Atta sexdens* and *Atta sexdens* fungal gardens (Fig. 3). The bacterial orders driving this difference in the bacterial community are Clostridiales, followed by Bacteroidales, and Propionibacteriales (Table 4). Clostridiales was found to drive the most difference between bacterial communities of *Atta sexdens* fungal gardens and natural habitat *A. sexdens*. Bacteroidales were found to be driving the second most amount of difference between bacterial communities, more abundant in the bacterial communities of fungal gardens. Propionibacteriales was found to be contributing the third most amount of difference between associated bacterial communities, much more abundant in natural habitat *A. sexdens*.

Clostridiales was found to be the most abundant in the bacterial community of *Atta sexdens* fungal gardens. It has been found to be highly abundant in whole *A. sexdens* workers (Vieira et al. 2017) and is highly abundant in specifically unveiled nests of *Apterostigma dentigerum* ants (González et al. 2019). Across 21 different orders of insects, Clostridiales, associated phyla Firmicutes, has been found to be significant in abundance, contributing the second highest abundance to gut bacterial communities (Gales et al. 2018; Yun et al. 2014). The high abundance of Clostridiales in *Atta* sexdens fungal gardens suggests that more research needs to be conducted to further explore the relationship of this bacterial order and the bacterial communities of *Atta* fungal gardens.

Bacteroidales was the second most abundant bacterial order found within *Atta sexdens* fungal gardens. Bacteroidales has been documented to have high host specificity to animal clades, used as a marker for fecal source (Dick et al. 2005; Layton et al. 2006). It has been found in more than 10% abundance in *Atta* gut bacterial communities across various development stages (Zhukova et al. 2017) as well as in high abundance across multiple taxa of termites (Noda et al. 2009). The presence of these bacteria in *Atta* fungal gardens may then have resulted from mutualism to *Atta*. There have been no other findings of Bacteroidales in the bacterial community of *Leucoagaricus gongylophorus*. The high abundance of these bacteria found within *Atta* fungal gardens is puzzling and requires further investigation.

The third most abundant bacteria in the bacterial community of the fungi is Bacillales, a member of the Bacteroidetes phylum. The presence of this bacterium is unsurprising as the phyla of Bacteroidetes has been previously found to be associated with *Atta* fungal gardens and has been found in the midgut of *A. sexdens* queens and workers (Vieira et al. 2017). Scott et al. (2010) suggest that the bacterial community of the fungi is strongly correlated to a mutualistic function, yet found no significant correlation of fungal gardens to host ant genus when comparing between *Atta* and *Acromyrmex*. To understand the relationship between *Atta* and its mutualistic fungi, it is imperative for future research to be conducted to examine the results of dissimilarity in bacterial communities between the bacterial communities of other *Atta* species in this study and *Atta sexdens* in other conditions.

## Conclusion

The importance of host-microbe interactions of *Atta* spp. has been well established due to mutualism with fungi, *Leucoagaricus gongylophorus*, but the beta diversity of the bacterial community of the *Atta* genus still needs more investigation. Four dominant *Atta* spp*.* found in the southern Neotropics were compared, revealing that bacterial communities of *Atta* spp*.* are significantly different despite occupying the same ecological niche and being allopatric. This was shown in this study by a significant difference found between *A. sexdens* and *A. laevigata*. Bacterial communities of samples collected in their natural habitat were compared to bacterial communities of samples kept in laboratory conditions. Findings illustrate that keeping *Atta* spp. (*A. sexdens*) in laboratory conditions significantly impacts associated bacterial communities. Bacterial communities of pesticide-treated *A. sexdens* significantly differed from natural habitat *A. sexdens.* Surprisingly, bacterial communities of pesticide-treated *A. sexdens* did not significantly differ from *A. sexden*s kept in the laboratory, emphasizing the importance of collection conditions and future work to explore this unexpected similarity in associated bacteria. Using 16S rRNA amplicon sequencing to analyze variable host factors, this study is the first to establish that the associated bacterial communities of *Atta* spp*.* as transient, meaning that their bacterial communities are variable and have taxa that may not always be present in the host. Highly abundant bacterial orders associated with the *Atta* samples were Entomoplasmatales, Clostriales, and Bacilliales. Bacteroidales, Burkholderiales, Propionibacteriales, Corynebacteriales, Enterobacteriales, and Xanthomonadales were found to be transient, with variable associations in *Atta* bacterial communities. These bacteria were significantly different in composition and abundance when compared to lab-reared samples. Bacterial communities of mutualistic *Atta sexdens* fungal gardens samples were found to significantly differ from the bacterial communities of the symbiont, *A. sexdens*. Multiple factors can drive bacterial community diversity, especially in cases where the microbiota composition is more variable or transient, as observed in the *Atta* species studied here. By comparing the similarity between bacterial communities of samples, this study shows that there is significant dissimilarity between bacterial community composition and abundance of *Atta* spp. *A. sexden*s and *A. laevigata*, and *A. sexden*s fungal gardens and *A. sexden*s. Similarity was found between composition and abundance of bacterial communities of lab-reared and pesticide-treated *A. sexden*s. These results support a more variable or transient microbiota of *Atta* spp. and call for future research of bacterial communities of *Atta* spp. to explore why these results are present.

## Supplementary Information

Below is the link to the electronic supplementary material.

ESM 1 (DOCX 573 KB)

## Data Availability

All raw sequence data are publicly available in NCBI SRA accession number PRJNA556881 and study SAMN12373152.
