# Supplementary material for: Bacterial Diversity in Leaf-Cutter Ant Species: Host-Microbe Interactions and Environmental Effects
Source: Neotrop Entomol. 2026 Jun 11;55(1):59. doi: 10.1007/s13744-026-01386-7 (PMC13260043; doi:10.1007/s13744-026-01386-7)
Supplement: Supplementary file 1 — (DOCX 573 KB) [file 13744_2026_1386_MOESM1_ESM.docx]

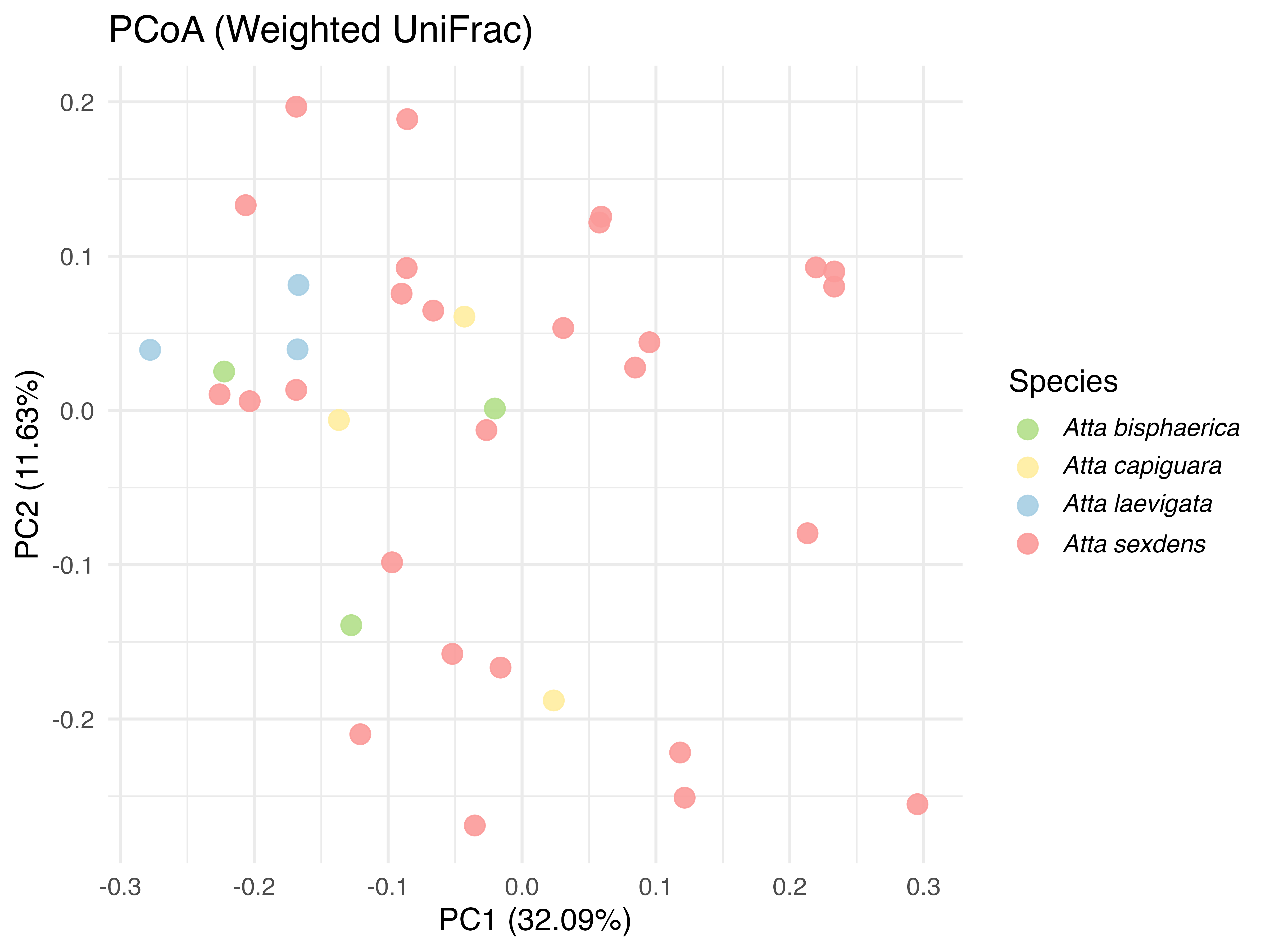


**Supplemental Figure 1.** Unifrac Weighted Principal Coordinate Analysis (PCoA) of all four *Atta* species (*Atta laevigata, Atta bisphaerica, Atta capiguara, Atta sexdens*) Including Non-Pesticide-Treated (Healthy) Lab-Harvested *Atta sexdens* with Unifrac weighted showing composition and abundance of *Atta* bacterial communities. Each dot represents the bacterial community composition of one sample. The closer the dots are together, the more similar their bacterial community composition. PERMANOVA Unifrac weighted, P-value = 0.003, Pseudo F = 3.306
